# Fusidic acid and clindamycin resistance in community-associated, methicillin-resistant *Staphylococcus aureus *infections in children of Central Greece

**DOI:** 10.1186/1471-2334-10-351

**Published:** 2010-12-13

**Authors:** George D Katopodis, Ioanna N Grivea, Angeliki J Tsantsaridou, Spyros Pournaras, Efi Petinaki, George A Syrogiannopoulos

**Affiliations:** 1 Department of Pediatrics, University of Thessaly, Medical School, Biopolis, 411 10 Larissa, Greece; 2 Department of Thoracic Surgery, University of Thessaly, Medical School, Biopolis, 411 10 Larissa, Greece; 3 Department of Microbiology, University of Thessaly, Medical School, Biopolis, 411 10 Larissa, Greece

## Abstract

**Introduction:**

In Greece, fusidic acid and clindamycin are commonly used for the empiric therapy of suspected staphylococcal infections.

**Methods:**

The medical records of children examined at the outpatient clinics or admitted to the pediatric wards of the University General Hospital of Larissa, Central Greece, with community-associated staphylococcal infections from January 2003 to December 2009 were reviewed.

**Results:**

Of 309 children (0-14 years old), 21 (6.8%) had invasive infections and 288 (93.2%) skin and soft tissue infections (SSTIs). Thirty-five patients were ≤30 days of age. The proportion of staphylococcal infections caused by a community-associated methicillin-resistant *Staphylococcus aureus *(CA-MRSA) isolate increased from 51.5% (69 of 134) in 2003-2006 to 63.4% (111 of 175) in 2007-2009 (*P *= 0.037). Among the CA-MRSA isolates, 88.9% were resistant to fusidic acid, 77.6% to tetracycline, and 21.1% to clindamycin. Clindamycin resistance increased from 0% (2003) to 31.2% (2009) among the CA-MRSA isolates (*P *= 0.011). Over the 7-year period, an increase in multidrug-resistant CA-MRSA isolates was observed (*P *= 0.004). One hundred and thirty-one (93.6%) of the 140 tested MRSA isolates were Panton-Valentine leukocidin-positive. Multilocus sequence typing of 72 CA-MRSA isolates revealed that they belonged to ST80 (n = 61), ST30 (n = 6), ST377 (n = 3), ST22 (n = 1), and ST152 (n = 1). Resistance to fusidic acid was observed in ST80 (58/61), ST30 (1/6), and ST22 (1/1) isolates.

**Conclusion:**

In areas with high rate of infections caused by multidrug-resistant CA-MRSA isolates, predominantly belonging to the European ST80 clone, fusidic acid and clindamycin should be used cautiously as empiric therapy in patients with suspected severe staphylococcal infections.

## Background

Since the late 1990's, an increasing number of reports from different parts of the world have described the occurrence of infections caused by community-associated methicillin-resistant *Staphylococcus aureus *(CA-MRSA) [1-5]. The presence of genes encoding for toxins such as Panton-Valentine leukocidin (PVL) appears to contribute to increased virulence of CA-MRSA isolates [6,7]. The vast-majority of CA-MRSA infections is skin and soft-tissue infections (SSTIs); however, certain cases can progress to invasive tissue infections and bacteremia.

Clindamycin has been used successfully in the treatment of both invasive and non-invasive CA-MRSA infections in children [8,9]. So, many clinicians use clindamycin as an empiric antimicrobial therapy for suspected staphylococcal infections. In addition, there is an increase in the use of antibiotics such as vancomycin, which may add further to the problem of antibiotic resistance in the hospital and the community [10].

In Greece, a PVL-producing CA-MRSA strain carrying the staphylococcal cassette chromosome (SCC) *mec *type IV and the accessory gene regulator (*agr*) allele 3, belonging to a clone C, identical to European CA-MRSA ST80, was initially identified in Patras in 1998 [11]. Following that observation, an increased incidence of CA-MRSA infections has been recognized among Greek children and adults [12].

In Greece, clindamycin, vancomycin, rifampin, and fusidic acid are the most commonly used non-β-lactam antistaphylococcal agents. In addition, quinolones are also being used, but almost exclusively in adults. Fusidic acid has been a common choice for empiric antistaphylococcal therapy, both parenterally and orally administered. In infections requiring parenteral therapy, it is recommended that fusidic acid should be administered in combination with another antistaphylococcal antibiotic [13]. Before the spread of CA-MRSA, a β-lactam was most often used in this combination.

The University General Hospital of Larissa (UGHL) serves as the academic, tertiary care referral center for the broader area of Central Greece. The aim of the present study was to evaluate a) the clinical manifestations of community-associated *S. aureus *infections in children who were examined at the outpatient clinics or hospitalized in the pediatric wards of the UGHL during a 7-year period and b) the possible changes in the phenotype and the genotype of the recovered MRSA isolates over the same time-period.

## Methods

### Patients

The medical records of children 0-14 years of age examined at the outpatient clinics or admitted to the pediatric wards of the UGHL, Central Greece, with community-associated staphylococcal infections from January 2003 to December 2009 were reviewed. In the present study, the research conformed to the Helsinki Declaration and to local legislation. The research protocol was approved by the Ethics Committee of the UGHL.

Community-associated infections were defined as those in which the *S. aureus *strain was isolated in a clinical specimen obtained within 48 hours from admission without hospitalization or surgery, indwelling catheter, prosthetic devices or positive MRSA culture within the preceding 1 year [2]. A standard preformed questionnaire was filled out for each patient including demographic data, type of infection-diagnosis, site of positive culture, surgical intervention, antibiotic treatment and outcome.

A case of invasive infection was defined by 1 or more of the following conditions: bacteremia, pneumonia, mastoiditis, lymphadenitis, septic arthritis, osteomyelitis, pyomyositis or another illness in which *S. aureus *was isolated from normally sterile body fluids. A diagnosis of staphylococcal pneumonia was made if the patient had radiographic abnormalities compatible with pneumonia and the blood or pleural fluid culture was positive for *S. aureus*. Infections involving the skin or soft tissue structures were regarded as SSTIs.

Patients were excluded if a diagnosis of *S. aureus *infection was made on the basis of positive *S. aureus *cultures obtained from the nose, axilla, or perineum or if they had a positive culture but no signs of disease. Patients with an orbital or otogenic infection were excluded if the *S. aureus *isolates were recovered from the swabs of the eye or ear drainage only [10].

### *S. aureus *identification and antimicrobial susceptibility testing

The clinical microbiology laboratory of the UGHL isolated and identified *S. aureus *strains by standard procedures [12]. Antimicrobial susceptibility testing was performed using the disk diffusion method according to Clinical and Laboratory Standards Institute (CLSI) recommendations and definitions [14,15] for oxacillin, fusidic acid, erythromycin, clindamycin, gentamicin, ciprofloxacin, tetracycline, trimethoprim-sulfamethoxazole, rifampin, vancomycin, linezolid, and mupirocin (BBL, Becton Dickinson, Le Pont de Claix, France). MIC to oxacillin was determined by use of an E-test (AB Biodisk, Solna, Sweden) according to the manufacturer's recommendations and was interpreted according to CLSI guidelines [15]. In addition, MIC to mupirocin of the mupirocin-resistant isolates was determined by use of an E-test. For fusidic acid, where CLSI does not provide disk susceptibility breakpoints, the required diameters for susceptibility and resistance were ≥22 mm and <22 mm, respectively (10-μg disc) according to the European Committee on Antimicrobial Susceptibility Testing (EUCAST) clinical breakpoints [16].

For isolates that tested resistant to erythromycin but susceptible to clindamycin, a D-test was performed to detect inducible resistance to clindamycin. Among MRSA, multidrug resistance was defined as resistance to three or more non-β-lactam antimicrobial agents (ciprofloxacin, clindamycin, erythromycin, gentamicin, tetracycline, rifampin, fusidic acid, trimethoprim-sulfamethoxazole, vancomycin, and linezolid).

### Detection of genes encoding *S. aureus *virulence factors

One hundred and forty of the 180 CA-MRSA isolates and 55 of the 129 community-associated methicillin-susceptible *S. aureus *(CA-MSSA) isolates of different antibiotic resistance patterns were randomly selected for the PVL testing. DNA was extracted from these 195 *S. aureus *isolates and polymerase chain reaction was completed to detect the genes encoding the PVL production (*luk*-*S*-*PV *and *luk*-*F*-*PV*). Primers and polymerase chain reaction conditions have been reported previously [12]. *S. aureus *ATCC 49775 was used as a positive control for the PVL genes.

### Multilocus sequence typing (MLST)

Seventy-two MRSA isolates of different antibiotic resistance patterns were randomly selected and further analyzed by MLST as previously described [17].

### Statistical analysis

To assess the annual hospital admission rates, groups of patients, and collections of isolates during the 7-year study period, categorical parameters were compared using the χ^2 ^for trend. In assessing two groups, continuous parameters were compared using Mann-Whitney U test and categorical parameters were compared using Fisher's exact test. *P *< 0.05 was considered statistically significant. The statistical analysis was carried out with the software product SPSS version 13.0.

## Results

During the study period, 309 children with community-associated *S. aureus *infections were identified from the database by a computer-assisted laboratory-based surveillance and medical record review; 180 (58.3%) had an infection due to an MRSA isolate. One hundred fifty-one (48.9%) of the 309 children were inpatients.

### Characteristics of patients with MRSA and MSSA infections

Patients from whom an MRSA isolate was recovered were significantly younger than those with MSSA (Table 1). Among the hospitalized children with an invasive staphylococcal infection, the mean ± SD age was 7.4 ± 5.4 years for MSSA and 2.8 ± 3.8 years for MRSA infection (*P *= 0.035). Among the hospitalized children with SSTIs, the mean ± SD age was 3.6 ± 4.0 years for MSSA and 2.5 ± 3.0 years for MRSA infection (*P *= 0.072).

**Table 1:** Characteristics of 309 patients with community-associated *Staphylococcus aureus* infections

Characteristic	MSSA	MRSA	*P*
	n = 129	n = 180	
Age, years			
All patients			
median (range)	4.5 (0.02 - 14)	1.7 (0.02 - 14)	
mean ± SD	4.8 ± 4.0	2.7 ± 3.0	< 0.001
Inpatients	52	99	
median (range)	2.4 (0.02 - 14)	1.2 (0.02 - 14)	
mean ± SD	4.3 ± 4.5	2.5 ± 3.1	0.005
Outpatients	77	81	
median (range)	5.0 (0.04 - 13)	2.0 (0.04 - 11)	
mean ± SD	5.2 ± 3.5	3.1 ± 2.9	< 0.001
Male gender	70 (54.3)^a^	88 (48.9)	0.359
Season			
June - November	97 (75.2)	125 (69.4)	0.306
Hospitalization	52 (40.3)	99 (55.0)	0.011

^a^number in parentheses is percent, unless otherwise indicated.

**Figure 1:**
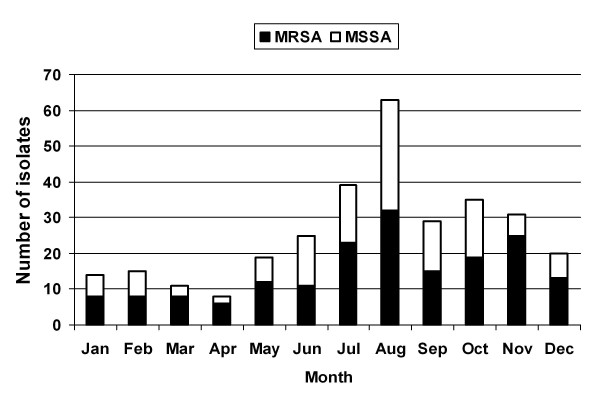
**Monthly distribution of community-associated *Staphylococcus aureus *infections in children at the University General Hospital of Larissa from 1 January 2003 through 31 December 2009**.

The monthly distribution of community-associated *S. aureus *isolates during the 7-year study period demonstrated an increasing number of isolates during the summer and autumn (Figure 1).

### Infections caused by CA-MRSA and CA-MSSA strains

Of the 309 *S. aureus *infections, 35 (11.3%) occurred during the first month of life and 10 (3.2%) during the 2nd and 3rd month (Table 2). These 45 young infants with *S. aureus *infection were previously healthy, were born at ≥37 weeks' gestation, and presented with a *S. aureus *infection after nursery discharge. MRSA was recovered from 23 (65.7%) of the 35 neonates with no history of admission to NICU or other hospitalizations since birth and no surgeries. In very young infants, MRSA infections were significantly more common than the MSSA ones (*P *= 0.0004).

**Table 2:** Community-associated *Staphylococcus aureus* infections during the first 90 days of life

Feature	Age				
	
	1-30 days		31-60 days	61-90 days	
	n = 35		n = 5	n = 5	
Age, days					
Median (range)	14 (7 - 30)		46 (32 - 60)	82 (63 - 90)	
Mean ± SD	15.6 ± 5.3		46.6 ± 10.0	77.6 ± 12.5	
Male gender	17 (48.6)^a^		3 (60.0)	2 (40.0)	
**Infection**	**MSSA**	**MRSA**	**MRSA**	**MSSA**	**MRSA**
	**n = 12**	**n = 23**	**n = 5**	**n = 2**	**n = 3**
Pneumonia with empyema	0	2 (8.7)	1 (20.0)	1 (50.0)	1 (33.3)
Septicemia	1 (8.3)	0	0	0	0
Pustulosis	5 (41.7)	11 (47.8)	1 (20.0)	0	2 (66.7)
Impetigo	2 (16.7)	0	1 (20.0)	0	0
Furuncle	1 (8.3)	2 (8.7)	1 (20.0)	1 (50.0)	0
Abscess	1 (8.3)	1 (4.3)	0	0	0
Cellulitis	0	1 (4.3)	0	0	0
Mastitis ± abscess	1 (8.3)	6 (26.1)	0	0	0
Omphalitis	1 (8.3)	0	0	0	0
Paronychia	0	0	1 (20.0)	0	0

^a^number in parentheses is percent, unless otherwise indicated.

Two hundred sixty-four (85.4%) of the 309 children with *S. aureus *infection were older than 90 days of life. The sites and types of invasive and non-invasive infections after the 3rd month of life appear in Table 3. Over the 7 years of the study, all the children with invasive *S. aureus *infections survived.

**Table 3:** Clinical features of patients >3 months old with community-associated *Staphylococcus aureus* infections

Type of infection	MSSA	MRSA	*P*
	n = 115	n = 149	
Invasive			
Arthritis	3 (2.6)^a^	1 (0.7)	0.321
Osteomyelitis	2 (1.7)	0	0.189
Pyomyositis/osteomyelitis with bacteremia	1 (0.9)	1 (0.7)	0.999
Bursitis with bacteremia	1 (0.9)	0	0.436
Pneumonia	0	2 (1.3)	0.506
Periorbital cellulitis with bacteremia	0	1 (0.7)	0.999
Soft tissue infection with bacteremia	0	2 (1.3)	0.506
Cervical lymphadenitis	0	1 (0.7)	0.999
			
SSTIs			
Impetigo	68 (59.1)	27 (18.1)	< 0.001
Folliculitis	1 (0.9)	3 (2)	0.635
Unspecified pustular lesions	11 (9.6)	8 (5.4)	0.232
Ecthyma	2 (1.7)	0	0.189
Furuncle	9 (7.8)	55 (36.9)	< 0.001
Abscess	4 (3.5)	22 (14.8)	0.003
Cellulitis	7 (6.1)	19 (12.7)	0.095
Paronychia	2 (1.7)	4 (2.7)	0.700
Mastitis	0	1 (0.7)	0.999
Infected wound	4 (3.5)	2 (1.3)	0.409

^a^number in parentheses is percent

Of the total 180 CA-MRSA isolates, 168 (93.3%) were recovered from children with SSTIs and 12 (6.7%) from children with invasive infections. Of the 129 CA-MSSA isolates, 120 (93.0%) were associated with SSTIs and 9 (7.0%) with invasive infections.

Among all the children with SSTIs, 51.8% (87 of 168) of those with CA-MRSA isolates and 35.8% (43 of 120) of those with CA-MSSA isolates were admitted to the hospital (*P *= 0.008). MRSA isolates accounted for a significantly higher proportion of SSTIs, such as furunculosis and abscesses, compared to MSSA isolates (*P *< 0.005).

**Figure 2:**
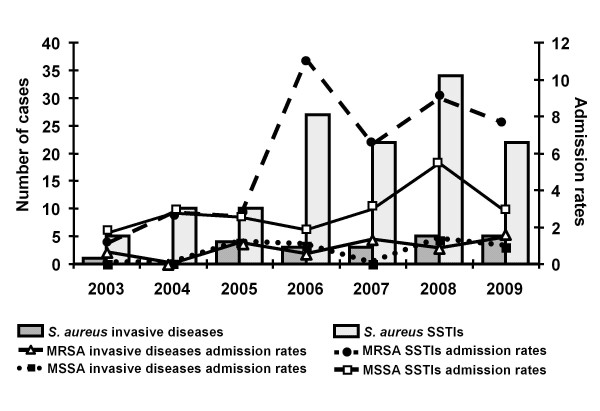
**Number of cases and admission rates (number of cases per 1000 hospital admissions) of community-associated *Staphylococcus aureus *infections among hospitalized pediatric patients**.

The annual distribution of cases and admission rates (number of cases per 1000 hospital admissions) during the 7-year study period are shown in Figure 2. The annual admission rates of community-associated infections caused by MRSA increased yearly (x^2 ^for trend, *P *= 0.0004). This change reflected mainly the increase in the rate of admissions for MRSA SSTIs (*P *= 0.0004). The yearly increase of the annual admission rates of community-associated infections caused by MSSA isolates was of a lesser degree compared to those due to MRSA (x^2 ^for trend, *P *= 0.053).

### Antibiotic susceptibility

**Figure 3:**
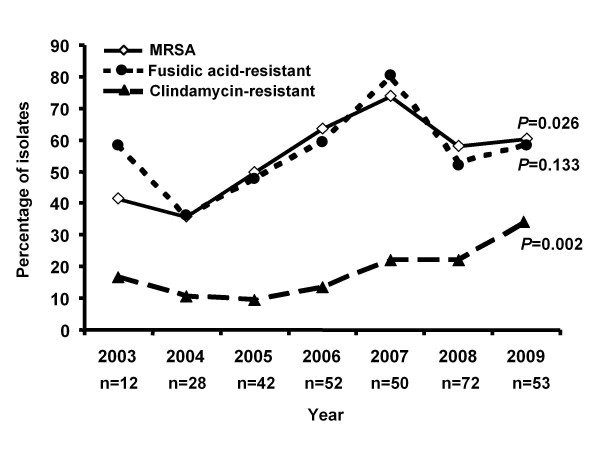
**Percentage of *Staphylococcus aureus *isolates with resistance to methicillin, fusidic acid, and clindamycin during the 7-year study period**.

Over the 7 years of the study, a significant increase in the resistance to methicillin and clindamycin was noted among the 309 *S. aureus *isolates (Figure 3). The proportion of staphylococcal infections caused by a CA-MRSA isolate increased from 51.5% (69 of 134) in 2003-2006 to 63.4% (111 of 175) in 2007-2009 (*P *= 0.037). Among the CA-MRSA isolates, 21.1% exhibited resistance to clindamycin, which specifically increased from 0% (2003) to 31.2% (2009) (x^2 ^for trend, *P *= 0.011). In addition, a trend of rise in the rate of clindamycin resistance was observed among the CA-MSSA (*P *= 0.072). The percentage of clindamycin resistance in MRSA isolates from 2007 to 2009 has been 23.8-31.2% and that of MSSA isolates 15.4-38.1%. The highest rate of clindamycin resistance among the MRSA and MSSA isolates was observed in 2009.

Resistance to fusidic acid was observed in 88.9% (160 of 180) of MRSA isolates and 22.5% (29 of 129) of MSSA (*P *< 0.001). Tetracycline resistance was noted in 77.6% (139 of 179) of MRSA isolates and 20.1% (26 of 129) of MSSA (*P *< 0.001). Resistance to gentamicin was noted in 3.9% (7 of 180) of MRSA isolates and none of the 129 MSSA ones (*P *= 0.044). Resistance to mupirocin was observed in 2 (0.98%) of the 205 tested *S. aureus *isolates and both of them were MSSA. In addition, there was only one MSSA isolate resistant to trimethoprim-sulfamethoxazole and another one to ciprofloxacin. All *S. aureus *isolates were susceptible to rifampin, vancomycin, and linezolid.

During the 7-year study period, a significant increase in multidrug-resistant (MDR) CA-MRSA isolates was observed (x^2 ^for trend, *P *= 0.004). Among the MRSA isolates, MDR increased from 0% (2003) to 31.2% (2009) (x^2 ^for trend, *P *= 0.011). Overall, 38 (21.1%) of 180 MRSA isolates were MDR compared with 9 (7.0%) of 129 MSSA isolates (*P *= 0.001). The rate of MDR MRSA isolates increased mainly during the second part of the present study, ie between 2007 and 2009, and compared to the rate during the years 2003-2006 reached a statistically significant level in the group of children with SSTIs (Table 4). An MDR CA-MRSA isolate was recovered from 20 (13.2%) of 151 inpatients and from 18 (11.4%) of 158 outpatients (*P *= 0.729).

**Table 4:** Characteristics of *Staphylococcus aureus* isolates according to the study period and the type of infection

Type of infection, isolate	2003-2006	2007-2009	*P*
	n = 134	n = 175	
Invasive	8	13	
MRSA	4 (50.0)^a^	8 (61.5)	0.673
MDR-MRSA	0	3 (23.1)	0.261
PVL-positive/MRSA tested	4/4 (100)	7/7 (100)	0.999
			
SSTIs	126	162	
MRSA	65 (51.6)	103 (63.6)	0.054
MDR-MRSA	9 (7.1)	26 (16.0)	0.028
PVL-positive/MRSA tested	50/57 (87.7)	70/72 (97.2)	0.430

^a^number in parentheses is percent

### Molecular analysis of *S. aureus *isolates

PVL-positive were 131/140 (93.6%) of the MRSA and 7/55 (12.7%) of MSSA isolates. Among the tested MRSA isolates, all 11 isolates associated with invasive infections were PVL-positive as well as 120/129 (93%) of the isolates that caused SSTIs. In contrast, only 2 (25%) of the 8 tested MSSA isolates associated with invasive infections were PVL-positive as well as 5 (10.6%) of the 47 MSSA isolates from SSTIs.

MLST analysis of the 72 tested MRSA isolates showed that they belonged to ST80 (n = 61), ST30 (n = 6), ST377 (n = 3), ST22 (n = 1), and ST152 (n = 1). Resistance to fusidic acid was observed in ST80 (58/61), ST30 (1/6), and ST22 (1/1) isolates. Among the ST80 MRSA isolates, 6 resistance patterns were observed; the two most common patterns exhibited resistance to oxacillin, fusidic acid, and tetracycline (n = 37) and oxacillin, fusidic acid, tetracycline, erythromycin, and clindamycin (n = 11). Of the 61 tested ST80 MRSA isolates, 16 (26.2%) were resistant to clindamycin expressing the inducible type of resistance. PVL-positive were mainly isolates belonging to ST80 and ST377. Of the 56 ST80 MRSA isolates that were tested for the PVL production, 54 (96.4%) were PVL-positive. In addition, all three ST377 isolates carried PVL genes. Only 2 MSSA isolates were analyzed by MLST; one belonged to ST152 and the other to ST489.

## Discussion

In Central Greece over the 7-year study period, MRSA strains have widely spread in the community and since 2005 account for >50% of all *S. aureus *isolates recovered from children with community-associated infections. These MRSA isolates are often MDR, exhibiting resistance to fusidic acid, tetracycline, erythromycin, and clindamycin. It appears to be a sustained phenomenon rather than a transient situation. In Greece, the excessive use of antibiotics may have contributed to this high frequency of MRSA isolates.

In our area, 85% of the tested MRSA isolates belonged to the European ST80. This finding suggests that the increase in MRSA infections is associated with the predominance and spread of a single clone in the community, which is the European ST80 [18,19]. Recent reports document the identification of the European ST80 clone also in Algeria [5], Tunisia [20], and Southern Israel [21].

It is not known why ST80 is so capable of rapidly spreading in a community or why it causes infections seemingly more readily than other *S. aureus *clones. The molecular analysis revealed that 93.6% of the MRSA isolates from Central Greece were PVL-positive. The suggested role of PVL in enhancing the organism's ability to spread rapidly or to increase severity of invasive infections is under investigation [7,22]. In addition, other factors are currently also investigated. A recent study described a class of secreted staphylococcal peptides that have a remarkable ability to recruit, activate and subsequently lyse human neutrophils, thus eliminating the main cellular defense against *S. aureus *infection [23].

As a consequence of the wide spread of such isolates in the community, the number of cases of SSTIs and invasive infections due to MRSA has increased across all ages. Of the 309 infections, 11.3% occurred during the first month of life. This is in accordance with the literature from areas with high rate of CA-MRSA [24,25].

Among the studied children with SSTIs, the proportion of cases admitted to the hospital was significantly higher in the group of patients with an MRSA infection than in those infected by an MSSA isolate. The difference in hospitalization frequencies is consistent with the CA-MRSA infections being more severe than the MSSA infections. The identification and antibiotic susceptibilities of the isolates were not known at the time of admission.

Fusidic acid is considered to be a very active agent for *S. aureus *and is available in intravenous, oral and/or topical (skin and ophthalmic) preparations. It has been a good choice for parenteral therapy, preferably as combination treatment, in moderate/severe suspected staphylococcal infections. However, in the present 7-year study, fusidic acid-resistant isolates, MRSA or MSSA, accounted for invasive infections and SSTIs. Resistance to this antibiotic has also been detected in CA-MSSA [26-28] as well as CA-MRSA strains [18,29-32] from other European countries. Both clonal [26] and non-clonal [28] dissemination of fusidic acid-resistant *S. aureus *have been well documented to occur in the community.

In contrast to CA-MRSA isolates from other continents, those belonging to the European ST80 clone have been reported to frequently express reduced susceptibility to fusidic acid and commonly carry the plasmid-located *far*-*1 *(also known as *fusB*) gene encoding fusidic acid resistance [18,32]. It is notable that in some previous reports from Europe these isolates have been reported as intermediate to fusidic acid [18]. This is related to the fact that due to the nonexistence of CLSI breakpoints for fusidic acid the authors had applied the previous breakpoints of the French Society for Microbiology [33]. According to these breakpoints, MICs of 4 to 16 μg/ml were considered indicative of an isolate intermediate to fusidic acid, whereas the breakpoint for resistance was ≥32 μg/ml. In 2010, the EUCAST breakpoints have been accepted in Europe and MICs of ≥2 μg/ml are considered representing resistance to fusidic acid [16]. Recently, Jones et al. have evaluated the activity of fusidic acid against *S. aureus *isolates using broth microdilution, disk diffusion, and Etest methods and proposed ≤1 μg/ml (disk zone ≥22 mm) as breakpoints for susceptibility and ≥4 μg/ml (disk zone ≤19 mm) for resistance [34].

In Greece, despite the high prevalence of fusidic acid-resistant *S. aureus *isolates in the community, the use of oral and/or topical (skin and ophthalmic) preparations of fusidic acid continues to be extensive (authors' unpublished data). Parenterally administered fusidic acid is used at a lower scale, but yet attention should be paid to this, as it usually concerns patients with severe infections. The prolonged use of fusidic acid as topical monotherapy for chronic skin conditions appears to have resulted in the emergence of resistance among *S. aureus *isolates in some countries [35,36], making this agent less active both for topical and systemic therapy [35]. In areas with high rate of fusidic acid resistance, topical and oral or systemic fusidic acid monotherapy should be restricted, in order to prevent the further spread of fusidic acid-resistant *S. aureus *isolates [35]. Topical use of a valuable systemically active agent is best to be avoided [36]. High frequency of fusidic acid resistance may persist despite such restrictions and may represent the development of a fusidic acid-resistant *S. aureus *reservoir in the community.

Since the time that the increase of CA-MRSA infections was well appreciated, clindamycin, an antibiotic that is considered to be active against a high percentage of CA-MRSA and MSSA isolates, has been extensively used empirically, particularly in pediatric infections [8,37]. In areas with low rate of clindamycin resistance, clindamycin is the recommended empirical antistaphylococcal therapy for hospitalized pediatric patients with clinical entities likely caused by CA-MRSA, such as cutaneous abscesses or pneumonia with empyema. In parallel to the antistaphylococcal activity, clindamycin possesses the ability to suppress production of PVL by *S. aureus in vitro *at the translational (ribosomal) level [38]. In the present study, PVL was produced by most of the MRSA isolates.

According to our recent data, the rate of clindamycin resistance in MRSA isolates from 2007 to 2009 has been 23.8-31.2% and that of MSSA isolates 15.4-38.1%. With the increased rates of clindamycin resistance among CA-MRSA and CA-MSSA isolates in our area, clindamycin is not useful for empiric monotherapy treatment for suspected *S. aureus *invasive infections and severe SSTIs. Thus, one should reconsider the empirical treatment regimen, which, in our area, until recently consisted primarily of clindamycin or vancomycin. Empirical clindamycin treatment for suspected staphylococcal infections is not recommended in areas where the proportion of CA-MRSA isolates exceeds 10% to 15% [37]. Among staphylococcal infections due to isolates exhibiting inducible clindamycin resistance [39,40], the risk of treatment failure during clindamycin therapy is increased when there is a high bacterial inoculum [39].

In Greece, tetracycline resistance is common among the CA-MRSA isolates. This is a significant difference between the European ST80 clone and the USA300 [3]. Actually in the United States, oral tetracyclines, such as minocycline or doxycycline, are used as an alternative treatment for suspected *S. aureus *SSTIs in children 8 years of age and older [41]. Tetracyclines are particularly prescribed by the dermatologists. In Central Greece, trimethoprim-sulfamethoxazole and ciprofloxacin resistance appeared to be rare, whilst vancomycin, rifampin, and linezolid susceptibility was universal, reinforcing the utility of these compounds for the time being at least.

The increasing rate of CA-MRSA infections has important implications for patient management and for the selection of appropriate antimicrobial therapy. If possible, single treatment which is active against both MSSA and MRSA should be considered as an empirical therapy in patients with staphylococcal infections. However, caution should be paid in monotherapy, as well as in combination therapy, using antibiotics recently recognized as having reduced activity against *S. aureus*. Currently in Central Greece, vancomycin, linezolid, or daptomycin, as monotherapy or in combination with rifampin or gentamicin, appear to be choices for empiric therapy of severe or difficult to treat possible staphylococcal infections. Vancomycin is inferior to nafcillin for the treatment of invasive MSSA infections including bacteremia and endocarditis. Some experts have proposed nafcillin or another penicillinase-resistant penicillin in combination with an agent active against MRSA isolates, such as vancomycin, for critically ill patients [37]. If a penicillinase-resistant penicillin is used as an empiric combination therapy, attention should be paid to the selection of the second antibiotic. Trimethoprim-sulfamethoxazole is another treatment choice, particularly in SSTIs.

Mild/moderate SSTIs can be empirically treated with clindamycin or trimethoprim-sulfamethoxazole [41,42], either alone or in combination with rifampin. However, optimal management for pediatric CA-MRSA SSTIs has yet to be determined because of limited clinical studies and efficacy data. Drainage of the abscesses is the key to treatment and is helpful in isolating the causative pathogen. A number of studies have shown primarily good outcomes in patients whose abscess is drained regardless of the antibiotics administered [43,44].

There were several possible limitations to our study. This was a retrospective study with its inherent limitations in data availability. We did not have detailed information on maternal infection history or that of other family members, especially for the very young infants. Additionally, although we tried to classify the infections as accurately as possible, a small number of patients with SSTIs which was mainly described in the charts as pyodermatitis, was classified as "unspecified pustular lesions". However, this does not influence the statistical analysis for impetigo. Infections classified as impetigo were clearly described with this diagnosis in the medical records. Finally, the molelular analysis of the isolates was based on MLST typing of representative isolates. Pulse-field gel electrophoresis could have provided additional information on the clonal relationship of the isolates.

Because antimicrobial resistance continues to evolve, it is important to continue monitoring *S. aureus *infections in our area. This provides valuable data on resistance trends and contributes to more effective treatment recommendations for regional and national use. Physicians caring for children throughout Europe and the world are likely to face the same problem with CA-MRSA that we have described in this report, as the ST80 or other CA-MRSA clones are introduced into their communities.

## Conclusions

We have described the epidemiology, clinical manifestations, and antibiotic resistance of the causative agents in pediatric community-associated *S. aureus *infections in Central Greece. We found that MRSA is increasing as a cause of skin and soft tissue infections, as well as of the invasive ones, among all ages. In our area, the sustained phenomenon of the wide spread in the community of MRSA strains is predominantly due to isolates belonging to the European ST80. Among the MRSA isolates, 88.9% were resistant to fusidic acid and 77.6% to tetracycline. The percentage of clindamycin resistance in MRSA isolates from 2007 to 2009 has been 23.8-31.2% and that of MSSA isolates 15.4-38.1%. In areas with high rate of infections caused by multidrug-resistant CA-MRSA isolates, predominantly belonging to the European ST80 clone, fusidic acid and clindamycin should be used cautiously as empiric therapy in patients with suspected severe staphylococcal infections.

## Competing interests

The authors declare that they have no competing interests.

## Authors' contributions

GDK, ING, and GAS conceived and designed the study. GDK wrote the first draft of the paper and other coauthors contributed to the final draft. GDK and ING were responsible for conducting the study and managing the data. GDK conducted the statistical analyses and the interpretation of data. Others participated in data analysis and data interpretation. All authors read and approved the final manuscript.

## Pre-publication history

The pre-publication history for this paper can be accessed here:

http://www.biomedcentral.com/1471-2334/10/351/prepub
